# Loneliness in Brazil

**DOI:** 10.1590/0102-311XEN117625

**Published:** 2025-07-25

**Authors:** Karla Giacomin

**Affiliations:** 1 Secretaria Municipal de Saúde, Belo Horizonte, Brasil. Secretaria Municipal de Saúde Belo Horizonte Brasil

“*We are all in the same boat, alone*” (Irish saying).

Loneliness is a complex, multifaceted phenomenon that has become a growing concern on the global level. Countries such as the United Kingdom and Japan have created “Ministries of Loneliness” to develop strategies and public policies focused on combating isolation and encouraging community ties. The World Health Organization (WHO) ^1^ has warned of the “*loneliness epidemic*”, which threatens public health, with harmful impacts on physical and mental health comparable to those of smoking, obesity, and a sedentary lifestyle.

Loneliness worsened throughout the world with the COVID-19 pandemic, as social distancing limited face-to-face human interactions. To address the loneliness epidemic, the WHO has created the Commission on Social Connection (2024-2026), which promotes social connectedness as a global health priority. In June 2025, this commission is expected to launch a report with proposals for a global agenda on social connection ^1^.

Social isolation is defined as a lack of social contact and relationships, whereas loneliness regards the subjective feeling of being alone. This feeling arises from the difference between desired and perceived levels of interpersonal relationships, leading to a sense of not belonging to a social group, and has negative emotional and behavioral implications ^1^^,^^2^. Being alone experienced in a positive, voluntary way is denominated “solitude”.

Loneliness constitutes both a global and local issue, wherein social and economic determinants significantly contribute to its development and consequences. Faced with a problem of a global scope, each country will be urged to review behaviors and actions that can be taken individually or on the community level to identify and deal with loneliness ^1^ with an integrated vision, encouraging the attentive observation of healthcare providers for signs of disconnection and promoting public policies that strengthen community ties, inclusion, and social cohesion.

The paper *Loneliness in Brazil: A Silent Threat to Public Health*^3^, by Hisrael Passarelli-Araujo, analyses this phenomenon and investigates how the Brazilian Unified National Health System (SUS, acronym in Portuguese) addresses loneliness through community-based interventions and integrated healthcare strategies. The discussion on this issue is timely, as loneliness has been shown to be a problem of considerable proportions in Brazil, with the country often appearing at the top of global rankings of loneliness, whose multifaceted causes reflect both global trends and local particularities ^2^^,^^4^.

Passarelli-Araujo examined the difficulties in measuring loneliness, considering its subjective nature and the various factors that exert an influence on how individuals perceive and report this experience. In Brazil, the assessment of loneliness is particularly complicated by limited resources and infrastructure. However, the most recent *Brazilian National Health Survey* conducted in 2019 and the *Brazilian Longitudinal Study of Aging* included questions on loneliness, health status, lifestyle, and social support.

SUS has the potential to address loneliness through community-based preventive healthcare models. However, the numerous delays decrease its effectiveness. Passarelli-Araujo highlights financial constraints that limit the ability of the SUS to expand services, invest in infrastructure, and staff healthcare teams, especially primary care units, family health teams, and mental health services. The imbalance in patient-to-professional ratios, the uneven distribution of healthcare providers across regions, and the fragmented approach to care within the universal healthcare system make addressing loneliness a complicated issue. Moreover, healthcare providers often lack training to identify signs of loneliness and its effect on health outcomes. The cultural stigma surrounding mental health also contributes to the underreporting and underdiagnosis of loneliness and related mental health conditions. All these factors impede the establishment of the integrated care needed for managing complex issues such as loneliness, which involves physical, emotional, and social aspects.

Moreover, Brazil is facing rapid, profound demographic changes, such as the aging of the population, changes in the composition of families, migration, and urbanization, which aggravate social isolation in various groups, including adolescents and older adults. Individualism, political polarization, and radicalization undermine dialogue and democracy, thus weakening social and family ties and contributing to loneliness. Austerity, poverty, and prejudices (e.g., racism, ageism, homophobia, and xenophobia) exacerbate the phenomena of inequality and social exclusion, thus intensifying feelings of loneliness. Family and school violence is also a contributing factor among Brazilian adolescents ^5^.

In older adults, loneliness is strongly associated with the development of anxiety, depression, and stress ^1^^,^^2^. Moreover, loneliness and social isolation increase the risk of dementia and cognitive decline, including Alzheimer’s disease ^6^. Lonely individuals have an increased risk of multi-morbidities (cardiovascular disease, hypertension, diabetes, infection, stroke, sleep disorders, chronic pain, etc.) as well as the worsening of preexisting health conditions ^7^.

Raising awareness on the impacts of loneliness and promoting an integrated look at the problem are crucial to building a more just, supportive, and humanly connected society. Coexistence projects, urban programs, and intergenerational activities can strengthen human connections and reduce social and gender inequalities that reinforce the prejudices that maintain individuals isolated from each other. To reduce loneliness, it is necessary to encourage and strengthen initiatives and solutions on the governmental, community, and individual levels that promote solidarity and value cultural and intergenerational programs to generate social cohesion, thus favoring community trust and resilience.
