# Supplementary material for: Loneliness in Brazil
Source: Cad Saude Publica. 2025 Jul 25;41(7):e00117625. doi: 10.1590/0102-311XEN117625 (PMC12334170; doi:10.1590/0102-311XEN117625)
Supplement: Translation

# Translation: Solidão no Brasil

**DOI:** 10.1590/0102-311XPT117625

**Authors:** Karla Giacomin

**Affiliations:** 1 Secretaria Municipal de Saúde, Belo Horizonte, Brasil.

“*Todo mundo está no mesmo barco, sozinho*” (ditado irlandês).

A solidão é um fenômeno complexo e multifacetado que vem se tornando uma preocupação crescente em nível global. Países como o Reino Unido e o Japão já criaram “Ministérios da Solidão” para desenvolver estratégias e políticas públicas focadas no combate ao isolamento e no incentivo de laços comunitários. A Organização Mundial da Saúde (OMS) ^1^ alertou para a “*epidemia de solidão*”, que ameaça a saúde pública, com impactos deletérios para a saúde física e mental comparáveis aos do tabagismo, da obesidade e do sedentarismo.

A solidão piorou em todo o mundo com a pandemia de COVID-19, pois o isolamento social limitou as interações humanas presenciais. Para enfrentar a epidemia de solidão, a OMS criou a Comissão sobre Conexão Social (2024-2026), que promove a conectividade social como prioridade global de saúde. Em junho de 2025, espera-se que essa comissão lance um relatório com propostas para uma agenda global sobre a conexão social ^1^.

O isolamento social se define como a falta de contato e relacionamentos sociais, enquanto a solidão se refere ao sentimento subjetivo de estar sozinho. Esse sentimento surge da diferença entre os níveis desejados e percebidos de relacionamentos interpessoais, levando a uma sensação de não pertencer a um grupo social, e tem implicações emocionais e comportamentais negativas ^1^^,^^2^. Estar sozinho, vivenciado de forma positiva e voluntária, é denominado “soledade”.

A solidão representa um problema tanto global e quanto local, no qual os determinantes sociais e econômicos contribuem significativamente para seu desenvolvimento e suas consequências. Diante de um problema de alcance global, cada país será estimulado a rever comportamentos e ações que podem ser adotados individualmente ou em nível comunitário para identificar e lidar com a solidão ^1^ com uma visão integrada, incentivando a observação atenta por parte dos profissionais de saúde aos sinais de desconexão e promovendo políticas públicas que fortaleçam os laços comunitários, bem como a inclusão e s coesão social.

O artigo *Loneliness in Brazil: A Silent Threat to Public Health* [Solidão no Brasil: Uma Ameaça Silenciosa à Saúde Pública] ^3^, de autoria de Hisrael Passarelli-Araujo, analisa esse fenômeno e investiga como o Sistema Único de Saúde (SUS) brasileiro aborda a solidão por meio de intervenções comunitárias e estratégias integradas de saúde. A discussão sobre essa questão é oportuna, visto que a solidão constitui um problema de grandes proporções no Brasil e o país frequentemente está no topo dos rankings globais de solidão, cujas causas multifacetadas refletem tanto tendências globais quanto particularidades locais ^2^^,^^4^.

Passarelli-Araujo examina as dificuldades em mensurar a solidão ao considerar sua natureza subjetiva e os diversos fatores que influenciam a forma como as pessoas percebem e relatam essa experiência. No Brasil, a avaliação da solidão é particularmente complicada pelos recursos e infraestrutura limitados. No entanto, a última *Pesquisa Nacional de Saúde* (PNS 2019) e o *Estudo Longitudinal da Saúde dos Idosos Brasileiros* (ELSI-Brazil) incluíram perguntas sobre solidão, estado de saúde, estilo de vida e apoio social.

O SUS tem o potencial de enfrentar a solidão por meio de modelos de saúde preventiva baseados na comunidade. No entanto, os inúmeros atrasos diminuem sua eficácia. Passarelli-Araujo destaca as restrições financeiras que limitam a capacidade do SUS de ampliar serviços, investir em infraestrutura e contratar equipes de saúde, especialmente nas unidades de atenção básica, nas equipes Estratégia Saúde da Família e nos serviços de saúde mental. O desequilíbrio na proporção de pacientes por profissional, a distribuição desigual de profissionais de saúde entre as regiões e a abordagem fragmentada da atenção dentro do SUS tornam o enfrentamento da solidão uma questão complexa. Além disso, os profissionais de saúde frequentemente carecem de treinamento para identificar os sinais da solidão e seus efeitos nos desfechos de saúde. O estigma cultural em torno da saúde mental também contribui para a subnotificação e o subdiagnóstico da solidão e das condições de saúde mental relacionadas. Todos esses fatores impedem o estabelecimento do cuidado integrado necessário para o manejo de questões complexas como a solidão, que envolve aspectos físicos, emocionais e sociais.

Ademais, o Brasil enfrenta mudanças demográficas rápidas e profundas, como o envelhecimento populacional, mudanças na composição familiar, migração e urbanização, que agravam o isolamento social em vários grupos, incluindo nos adolescentes e nos idosos. O individualismo, a polarização política e a radicalização minam o diálogo e a democracia, enfraquecendo os laços sociais e familiares e contribuindo para a solidão. A austeridade, a pobreza e os preconceitos (por exemplo, racismo, discriminação por idade, homofobia e xenofobia) exacerbam os fenômenos de desigualdade e de exclusão social, intensificando os sentimentos de solidão. A violência familiar e escolar também contribui à solidão entre os adolescentes brasileiros ^5^.

Nas pessoas idosas, a solidão está fortemente associada ao desenvolvimento da ansiedade, da depressão e do estresse ^1^^,^^2^. Além disso, a solidão e o isolamento social aumentam o risco de demência e de declínio cognitivo, incluindo a doença de Alzheimer ^6^. Indivíduos solitários apresentam um risco aumentado de multimorbidades (doenças cardiovasculares, hipertensão, diabetes, infecções, derrames, distúrbios do sono, dor crônica e outras), bem como o agravamento de condições de saúde preexistentes ^7^.

A sensibilização da população sobre os impactos da solidão e a promoção de uma visão integrada do problema são cruciais para a construção de uma sociedade mais justa, solidária e conectada. Projetos de convivência, programas urbanos e atividades intergeracionais podem fortalecer as conexões humanas e reduzir as desigualdades sociais e de gênero que reforçam os preconceitos que mantêm os indivíduos isolados uns dos outros. Para reduzir a solidão, é necessário incentivar e fortalecer iniciativas e soluções nos níveis governamental, comunitário e individual que promovam a solidariedade e valorizem programas culturais e intergeracionais para gerar coesão social, favorecendo assim a confiança e a resiliência comunitária.

# Translation: Soledad en Brasil

**DOI:** 10.1590/0102-311XES117625

**Authors:** Karla Giacomin

**Affiliations:** 1 Secretaria Municipal de Saúde, Belo Horizonte, Brasil.

“*Todos estamos en el mismo barco, solos*” (refrán irlandés).

La soledad es un fenómeno complejo y polifacético que se ha convertido en una preocupación creciente a nivel global y también en Brasil. Países como el Reino Unido y Japón han creado “Ministerios de la Soledad” para desarrollar estrategias y políticas públicas enfocadas en combatir el aislamiento y fomentar los vínculos comunitarios. La Organización Mundial de la Salud (OMS) ^1^ ha alertado sobre la “*epidemia de soledad*” que amenaza la salud pública con impactos nocivos en la salud física y mental comparables a los del tabaquismo, la obesidad y el sedentarismo.

En todo el mundo, la soledad ha empeorado con la pandemia de la COVID-19 y el aislamiento social, el cual ha limitado las interacciones humanas cara a cara. Para hacer frente a la epidemia de soledad, la OMS ha creado una Comisión de Conexión Social (2024-2026) que busca promover la conectividad social como una prioridad de salud mundial. Todavía, en junio de 2025 aún se espera que esta Comisión publique un informe con propuestas para una agenda global de conexión social ^1^.

El aislamiento social se define como la falta de contactos y relaciones sociales, mientras que la soledad se refiere a la sensación subjetiva de estar solo. Este sentimiento surge de la diferencia entre los niveles deseados y percibidos de relaciones interpersonales, lo que lleva a una sensación de no pertenecer a un grupo social, y tiene implicaciones emocionales y conductuales negativas ^1^^,^^2^. Cuando la soledad se experimenta de manera positiva y voluntaria, es definida como solitud.

La soledad constituye un problema tanto a nivel local como global, en que los determinantes sociales y económicos contribuyen de manera significativa a su desarrollo y a sus consecuencias. Frente a una problemática de alcance global, se instará a cada país a revisar comportamientos y acciones que se pueden tomar de manera individual o comunitaria para identificar y enfrentar la soledad ^1^ con una visión integral, incentivando la mirada atenta de los profesionales de la salud por las señales de desconexión y promoviendo políticas públicas que fortalezcan los lazos comunitarios, la inclusión y la cohesión social.

El artículo *Loneliness in Brazil: A Silent Threat to Public Health* [La Soledad en Brasil: Una Amenaza Silenciosa para la Salud Pública] ^3^, de Hisrael Passarelli-Araujo, analiza este fenómeno y evalúa cómo el Sistema Único de Salud brasileño (SUS) aborda la soledad con intervenciones comunitarias y estrategias integradas de salud. Por lo tanto, la discusión propuesta en esta edición es muy oportuna, ya que, en Brasil, se ha demostrado que la soledad es un problema de proporciones considerables, con el país a menudo en los primeros puestos de los rankings mundiales de soledad, un fenómeno cuyas causas polifacéticas reflejan tanto las tendencias globales como las particularidades locales ^2^^,^^4^.

Passarelli-Araujo examinó las dificultades para medir la soledad, teniendo en cuenta su naturaleza subjetiva y los diversos factores que influyen en la forma en que los individuos perciben y reportan esta experiencia. En Brasil, la evaluación de la soledad resulta particularmente compleja debido a las limitaciones en recursos e infraestructura. Sin embargo, la última *Encuesta Nacional de Salud* (PNS 2019) y el *Estudio Longitudinal de Salud de los Ancianos Brasileños* (ELSI-Brasil) incluyeron preguntas sobre soledad, estado de salud, estilo de vida y apoyo social.

El SUS tiene el potencial de abordar la soledad mediante sus modelos de atención médica comunitaria y preventiva; sin embargo, muchos desafíos retrasan su efectividad. Passarelli-Araujo destaca las limitaciones financieras que limitan la capacidad del SUS para ampliar los servicios, invertir en infraestructura y dotar de personal a los equipos de salud -principalmente a las unidades de atención primaria, a los equipos de salud de la familia y a los servicios de salud mental. El desequilibrio en la proporción de pacientes por profesional, la distribución desigual de los profesionales de la salud en las regiones y el enfoque fragmentado de la atención dentro del SUS complican el abordaje de la soledad. Además, los profesionales de la salud a menudo carecen de la capacitación para identificar las indicaciones de soledad y su efecto en los resultados en salud. Además, el estigma cultural que rodea a la salud mental exacerba el subregistro y el infradiagnóstico de la soledad y las afecciones de salud mental relacionadas. Todos estos factores impiden una atención integral necesaria para el manejo de problemas complejos como la soledad, que involucra aspectos físicos, emocionales y sociales.

Por otra parte, Brasil se enfrenta a cambios demográficos profundos y rápidos, como el envejecimiento de la población, los cambios en la estructura familiar, la migración y la urbanización, los cuales aumentan el aislamiento social de diversos grupos, incluidos los adolescentes y los adultos mayores. El individualismo, la polarización política y la radicalización socavan el diálogo y la democracia, debilitando los lazos sociales y familiares y contribuyendo a la soledad. La austeridad, la pobreza y los prejuicios como el racismo, la discriminación por edad, la homofobia y la xenofobia crean desigualdad y exclusión, aumentando la soledad. La violencia familiar y escolar también contribuye para el aumento de la soledad entre los adolescentes brasileños ^5^.

En los adultos mayores, la soledad está fuertemente asociada con el desarrollo de ansiedad, depresión y estrés ^1^^,^^2^. La soledad y el aislamiento social también aumentan el riesgo de demencia y deterioro cognitivo, incluido el Alzheimer ^6^. Las personas solitarias tienen un mayor riesgo de multimorbilidades -enfermedades cardiovasculares, hipertensión, diabetes, infecciones, accidentes cerebrovasculares, trastornos del sueño, dolor crónico, entre otros- y del empeoramiento de condiciones de salud preexistentes ^7^.

Crear conciencia sobre los impactos de la soledad y promover una mirada integrada del problema son cruciales para construir una sociedad más justa, solidaria y humanamente conectada. Los proyectos de convivencia, los programas urbanos y las actividades intergeneracionales pueden fortalecer las conexiones humanas y reducir las desigualdades sociales y de género que refuerzan los prejuicios que insisten en aislarnos. Para reducir la soledad, es necesario fomentar y fortalecer iniciativas y soluciones, tanto gubernamentales como comunitarias e individuales, que promuevan la solidaridad e impulsen los programas culturales e intergeneracionales en generar cohesión social y favorecer la confianza y resiliencia comunitaria.
